# Novel CD47: SIRPα Dependent Mechanism for the Activation of STAT3 in Antigen-Presenting Cell

**DOI:** 10.1371/journal.pone.0075595

**Published:** 2013-09-20

**Authors:** Natan Toledano, Devorah Gur-Wahnon, Adi Ben-Yehuda, Jacob Rachmilewitz

**Affiliations:** Goldyne Savad Institute of Gene Therapy, Hadassah-Hebrew University Medical Center, Jerusalem, Israel; UTHealth Medical School, United States of America

## Abstract

Cell surface CD47 interacts with its receptor, signal-regulatory-protein α (SIRPα) that is expressed predominantly on macrophages, to inhibit phagocytosis of normal, healthy cells. This “don’t eat me” signal is mediated through tyrosine phosphorylation of SIRPα at the cytoplasmic ITIM motifs and the recruitment of the phosphatase, SHP-1. We previously revealed a novel mechanism for the activation of the STAT3 pathway and the regulation of human APC maturation and function that is based on cell:cell interaction. In this study, we present evidence supporting the notion that CD47:SIRPα serves as a cell surface receptor: ligand pair involved in this contact-dependent STAT3 activation and regulation of APC maturation. We show that upon co-culturing APC with various primary and tumor cell lines STAT3 phosphorylation and IL-10 expression are induced, and such regulation could be suppressed by specific CD47 siRNAs and shRNAs. Significantly, >50% reduction in CD47 expression abolished the contact-dependent inhibition of T cell activation. Furthermore, co-immunoprecipitation experiments revealed a physical association between SIRPα and STAT3. Thus, we suggest that in addition to signaling through the ITIM-SHP-1 complex that transmit an anti-phagocytotic, CD47:SIRPα also triggers STAT3 signaling that is linked to an immature APC phenotype and peripheral tolerance under steady state and pathological conditions.

## Introduction

Antigen-specific tolerance is believed to be critical for the prevention of autoimmunity and maintenance of immune homeostasis [1]. In addition to central tolerance by means of clonal deletion of self-reactive T cells, other mechanisms which take place in the periphery are also essential for tolerance maintenance. In the periphery, antigen presenting cells (APC), specifically dendritic cells (DC), are key regulators of immunity with the capacity to induce T cell activation as well as tolerance. Emerging data suggest that the functional activities of DC are mainly dependent on their state of activation and differentiation; that is, terminally-differentiated, mature DC can efficiently induce the development of T effector cells, whereas “immature” or “semi-mature” DC maintain peripheral tolerance [2]–[4]. The mechanism by which immature and semi-mature DC maintain peripheral tolerance is not clear, but it is well-established that they induce anergy in T cells, as well as induce a generation of T cells with regulatory properties or T cells that secrete immunomodulatory cytokines such as IL-10.

Although the molecular basis of APC tolerogenicity remains unclear, the transcription factor Signal Transducer and Activators of Transcription-3 (STAT3) has emerged as a key negative regulator of immunity, that is, STAT3 signaling is linked to APC immature phenotype, production of IL-10, and tolerance induction [5]. Importantly, targeted disruption of the STAT3 signaling pathway in mice leads to loss of T cell tolerance, highlighting the central role of STAT3 in maintaining peripheral tolerance, and the prevention of autoimmunity [5]. Moreover, previous studies have identified an immunomodulatory circuit initiated by STAT3 activation in tumor cells that drives anti-inflammatory cytokine production that, in turn, induces STAT3 activation within neighboring tumor infiltrating DC and converts them into regulatory cells [6]. Our study on the immunomodulatory properties of human mesenchymal stem cells (hMSC) and the way they inhibit T cell activation revealed an alternative mechanism for STAT3 activation. In this study, we demonstrated that hMSC inhibit T-cell activation through APC altered maturation and IL-10 secretion. Specifically, we have shown that the addition of APC (either monocytes or DC) to T cell-hMSC cultures was essential for T cell inhibition. Furthermore, this inhibitory activity was contact-dependent and resulted in the secretion of IL-10 [7]. We have also demonstrated that hMSC inhibitory activity was dependent on selective STAT3 activation in the APC (as demonstrated using intracellular staining and by inhibiting STAT3 activity within the APC) and, thereby, influenced their functional maturation [8]. Interestingly, we have further extended this observation to tumor cells and suggested that in the case of tumor-mediated APC modulation, there are two parallel mechanisms for the activation of STAT3, soluble cytokines versus cell:cell contact. In aggregate, we have identified a novel, contact-dependent mechanism for STAT3 activation by a previously unknown JAK2-dependent signaling pathway that precedes IL-10 secretion and is distinct from the well-established cytokine-mediated pathway [9].

This data suggested that, in at least certain cellular microenvironments, cell:cell interactions represent a novel way by which STAT3 signaling is activated, uncouple APC activation events, and consequently regulate immunity and tolerance. This novel mechanism also represented a new tumor escape mechanism that requires further investigation. Since this interaction occurs only when the cells come into productive contact, this mechanism can provide a molecular explanation for how the surrounding microenvironment influences APC maturation in tissues, in a much more focused way as compared to soluble systemic factors.

The CD47: signal-regulatory-protein α (SIRPα) pair caught our attention as a candidate receptor:ligand pair that may be involved in the contact-dependent induction of STAT3. CD47 (also called integrin-associated protein, IAP) is a cell surface transmembrane glycoprotein that is widely expressed on many cells of epithelial and mesenchymal origin, including hMSC, and is highly expressed on tumor cells, such as leukemia [10]. CD47 upregulation was recently found to serve as a mechanism for leukemia stem cells/progenitors to avoid phagocytosis [11], [12]. SIRPα (also known as CD172a or SHPS-1) is a transmembrane glycoprotein receptor that is expressed predominantly on myeloid and neuronal cells and has been linked to cell adhesion [13], [14]. SIRPα ligation, by its cognate ligand CD47, when used as a marker of ‘self’ [14]–[16] results in a negative signal that inhibits phagocytosis and prevents the phenotypic and functional maturation of DC [17], [18]. As a result, CD47 on live cells prevent their elimination by engaging SIRPα on phagocytes. Interestingly, it was also shown that inflammation is prolonged in CD47-deficient mice [19]. Reminiscent to our previous studies [7], [9], a recent study demonstrated that CD47:SIRPα ligation results in a partial block in DC maturation leading to a semimature DC phenotype [17]. Significantly, while SIRPα mostly generated a negative signal via immunoreceptor-based inhibition motifs (ITIMs) and the recruitment of phosphatases, predominantly SHP-1 [20], [21], other studies have demonstrated that SIRPα associate with JAK2 and that SIRPα ligation resulted in JAK/STAT activation in macrophages [22], [23].

Hence, in the present study we tested CD47:SIRPα as potential cell surface receptor:ligand pair responsible for the contact-dependent STAT3 activation. Our data demonstrate that ligation of SIRPα by CD47 expressed on the surface of neighboring cells induce STAT3 phosphorylation, IL-10 secretion and the induction of tolerogenic activity.

## Materials And Methods

### Cells

Buffy coat samples were obtained from Hadassah Blood Bank, under approval of Hadassah Medical Center Helsinki Ethics Committee. Peripheral blood mononuclear cells (PBMC) were purified from the venous blood of healthy donors by Ficoll-Hystopaque density gradient centrifugation (Sigma Aldrich, St Louis, MI, USA). CD14^+^ cells were isolated by negative selection using the RosetteSep™ enrichment cocktail (StemCell Technologies, Vancouver, Canada). For DC generation, CD14^+^ cells were plated in RPMI 1640 (Biological Industries) containing 1% autologous plasma, 0.1 µg/ml IL-4 and 0.1 µg/ml GM-CSF (PeproTech, Rocky Hill, NJ). Every 2 days 0.3 ml was removed and 0.5 ml media containing plasma and cytokines was added. By day 7, >90% of the cells were CD14^-^ and CD11c^+^ immature DCs. For JAK blockade freshly isolated CD14^+^ cells or DC were pre-incubated with JAK2 inhibitors, JSI-124 (Cucurbitacin I; Calbiochem,). hMSC were obtained from discarded bone tissues from patients undergoing total hip replacement surgeries, under approval of Hadassah Medical Center Helsinki Ethics Committee following a written informed consent. The hMSC were separated from other bone-marrow residing cells by plastic adherence and were then grown under tissue culture conditions, as previously described [8]. The cells were maintained in a low-glucose DMEM medium supplemented with 10% heat-inactivated fetal calf serum, 2 mM glutamine, and penicillin/streptomycin (Biological Industries, Beit-Haemek, Israel). Primary human fibroblasts obtained from skin tissues (under formal waiver as discarded tissue approved by Hadassah Medical Center Helsinki Ethics Committee) were provided by Dr. Zamir, Department of Surgery, Hadassah Hebrew-University Medical Center, Jerusalem, Israel. Human breast carcinoma (MCF-7), human Hepatoma (Hep3B) and HEK293 human embryonic kidney cell lines were obtained from the American Type Culture Collection (ATCC). Cells were cultured in DMEM with 10% heat-inactivated fetal calf serum, 2 mM glutamine, and penicillin/streptomycin (Biological Industries, Beit-Haemek, Israel) at 37°C and 5% CO_2_.

### Cell Lysis and Immunoblotting

The various cell types (2×10^5^) and APC (6×10^5^; either monocytes or DCs) alone, or the mixture of the two were incubated at 37°C in individual wells of 24-well plates (Corning, Corning, NY). After 2 hours, cell extracts were prepared using a lysis buffer (1% Nonidet P-40, 10 mM Tris-HCl (pH 7.8), 150 mM NaCl, 4 mM EDTA pH 8.0, 10 mM Na-pyrophosphate, 10 nM NaF, 1 mM PMSF, 4 mM Sodium Orthovanadate, 10 µg/ml Leupeptin, and 10 µg/ml Aprotinin) for 30–40 min on ice. Lysates were separated by electrophoresis on 10% SDS-PAGE gels and then transferred to PVDF membranes (Bio-Rad, Hercules, CA). The blots were probed with anti-phosphorylated STAT3 mAb (Tyr705, Cell Signaling, Danvers, MA) processed with ECL plus Western Blotting detection system (Amersham- Pharmacia Biotech) and exposed to Chemiluminescence BioMax Light Film (Kodak-Industries, Cedex, France). Following stripping, membranes were re-probed with anti STAT3 mAb (Cell Signaling).

### Cytokine Production

IL-10 and IL-27 secretion was determined in a 24 hour conditioned media of either monocytes, MCF-7 alone, or co-culture of the two using commercial ELISA (R&D Systems). Cultures containing 1×10^6^ PBMC were stimulated in individual wells of 24-well plates (Corning) with 10 ng/ml anti-CD3 mAb (OKT3; eBioscience, San Diego, CA, USA) and in the absence or presence of the various HEK293 transfectants (2×10^5^/well). Cells were stimulated for 72 hours and conditioned media were collected. IFN-γ levels in the conditioned media were assayed by ELISA (R&D Systems). Monocytes (0.6×10^6^/well) were plated in 24 well plates (Corning) and were stimulated with 10 ng/ml LPS (Sigma Aldrich) in the presence or absence of apoptoic cells (0.15×10^6^/well). Cells were incubated for 24 hours and conditioned media were collected. Medium was also collected from monocytes cultured alone. TNF-α and IL-1β levels in the conditioned media were assayed by ELISA (R&D Systems).

### Intracellular Staining and Flow Cytometry

The various cell types were stained with FITC-conjugated anti CD47 mAb (eBiosciense, San Diego, CA, USA). Monocytes and MCF-7 were cultured alone or mixed together for 24 hours, with the addition of 2 µM monensin for the last 5 hours of incubation. Subsequently, the cells were harvested, washed twice in PBS, fixed in 4% Paraformaldehyde for 30 minutes, washed twice in PBS 1% FCS, resuspended in 0.1% Saponin, 1% FCS/PBS and stained using PE-conjugated anti-cytokine IL-10 (Serotec, Oxford, UK) for 30 minutes. Cells were then washed twice in PBS and 1×10^4^ cells were analyzed on a FACS Calibure flow cytometer (Becton Dickinson, San Jose, CA) using the Cell Quest software.

### Immunoprecipitation

For immunoprecipitation, cell lysates of the various treatments (prepared as described above) were pre-cleared for 1 h with protein A-Sepharose beads (Sigma-Aldrich) and then incubated with the anti-SIRPα mAb (eBiosciense) for at least 1 h. Subsequently, protein A-Sepharose beads were added and incubated o/n with gentle rotation. Beads were then washed three times in PBS. Samples were resuspended in reducing SDS sample buffer, heated at 95°C for 10°min, and separated by SDS-PAGE. Proteins were transferred to PVDF membrane (Bio-Rad, Hercules, CA) for analysis by Western blotting as described above.

### Blocking CD47

To block CD47 expression, MCF-7 cells were transfected with either siRNA against human CD47 or control siRNA using Oligofectamine reagent, according to manufacturer protocol (Invitrogen, Carlsbad, CA). After 24 hours CD47 expression levels were determined using flow cytometric analysis. Cells were incubated in presence or absence of APC (either monocytes or DC) as described above. We tested several siRNA sequences. Significant knockdown was achieved using a combination of two siRNA duplexes: siRNA1∶5′-CUAUGAGACCCUUACGUGAUUGUUA-3′. siRNA-2∶5′-GCACAUGCAUCUUCUGUAUGGACAA-3′.

For blocking CD47: SIRPα interaction two anti-CD47 mAb were used (B6H12.2 and 2D3; eBioscience). The functional grade antibodies were added to MCF-7 cultures (10 µg/ml) 15 minutes prior to co-culturing them with APC.

### Apoptosis Induction

Cells were plated in a 24 well plate (Corning, Corning, NY) and were allowed to adhere over night, at 37°C and 5% CO2. The following day, apoptosis was induced by several methods: *Staurosporine*- cells were incubated with 0.5 µM Staurosporine (Sigma-Aldrich) for 1.5 h and then washed with fresh media. *γ-Irradiation*- cells were irradiated (10Gy in GammaCell 220 Excel, MDS Nordion). *Peroxide treatment*- medium was aspirated and cells were washed with PBS with Ca2^+^ and Mg2^+^ (Biological Industries). Cells were incubated with H_2_O_2_ (30%, Sigma, Steinheim, Germany) diluted in PBS to a final concentration of 200 µM, at 37°C. After 1 h, cells were washed and fresh medium was added. *Anti-FAS mAb*- cells were treated with 1 µg/ml of anti-human FAS mAb (MBL, Naka-ku Nagoya, Japan). The number of apoptotic cells in the cultures was determined at different time points following apoptosis induction using Annexin/PI kit (MBL) according to manufacturer instructions.

## Results

**Figure 1:**
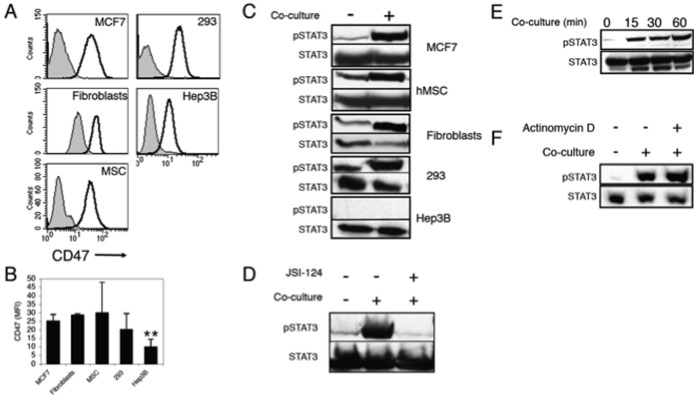
CD47 surface expression levels by various cell types and their corresponding ability to induce STAT3 phosphorylation in monocytes. (A) Different cell types were screened for surface CD47 expression using flow cytometric analysis. Grey histogram: isotype control. Black line: anti-CD47 staining. One representative experiment is shown. Comparable results were obtained in three independent experiments. (B) The average of three separate experiments is graphed as median fluorescence intensity (MFI) of CD47 staining. MFI was calculated by reducing the background isotype staining from the MFI value of anti-CD47 staining for each cell type. **- P≤0.001. (C) The various cell lines were co-cultured with monocytes for 2 h. Control cell extracts were obtained by incubating the cells and the monocytes separately and then mixing just before lysis. Cell extracts were subjected to SDS-PAGE and immunoblotting of anti-phosphorylated STAT3 (upper panels). Anti-STAT3 immunoblotting reveals relative amounts of protein in each lane (lower panels). Comparable results were obtained in at least two separate experiments for each cell type. Note: STAT3 phosphorylation in control lanes represents steady-state level of active STAT3 in the various cell lines. (D) Monocytes were either left untreated or treated with JSI-124 (10 µM) for 1 hour at 37°C, and then washed. Treated and untreated cells were then co-cultured with MCF7 and STAT3 phophorylation was determined as in B. (E) Monocytes were co-cultured with MCF7 cells for various time points and then lysed and analyzed as above. (F) Monocytes were co-cultured with MCF7 cells for 2 h in the absence or presence of 20 µM Actinomycin D, STAT3 phophorylation was determined as in B.

We screened several normal and cancerous primary cells and cell lines for their CD47 expression. These included the cancer cell lines MCF-7, Hep3B as well as the human embryonic kidney transformed cell line HEK293 [24]. We also used primary culture of hMSC and skin stromal primary fibroblasts. CD47 surface expression level was measured by flow cytometry analysis (Fig. 1A and B). Interestingly, all cells expressed equivalent levels of CD47 on their surfaces except for the Hep3B cell line that had significantly lower levels of CD47 in comparison to all other cells (Fig. 1A–B).

Using immuno-fluorescence intracellular staining, we have previously demonstrated that both tumor cells and hMSC induce STAT3 phosphorylation and activation in APC (both DC and monocytes) in a contact dependent manner [7]–[9]. To determine whether the level of CD47 expression correlates with the ability to induce STAT3 activation, we tested the ability of the various cell types to induce STAT3 phosphorylation following co-culture with monocytes. To that end, the various cells were incubated for 2 hours with monocytes as previously described [8]. Media was washed away just prior to adding the monocytes in order to avoid any effect of cell-derived soluble factor(s). The extracts from these various cell:monocyte co-cultures, as well as extracts from the two cell types cultured separately, were fractionated on SDS-PAGE and immunoblotted using anti-phosphotyrosine STAT3 mAb. Significant increase in STAT3 phosphorylation was detected in all cell types upon co-culture with monocytes except in Hep3B:monocyte cultures, in which CD47 expression was significantly lower (Fig. 1C). Notably, when monocytes were pre-incubated for one hour with JAK2 inhibitors, JSI-124, washed and then combined with MCF7, phophorylation of STAT3 was greatly reduced, suggesting that this phosphorylation event mainly originate in the monocytes (Fig. 1D). Moreover, STAT3 phosphorylation can be detected as early as 15 min after co-culture (Fig. 1E) and was not sensitive to protein transcription inhibitor, Actinomycin D, demonstrating that the STAT3 activation is not due to a secondary effect following cell:cell contact (Fig. 1F).

**Figure 2:**
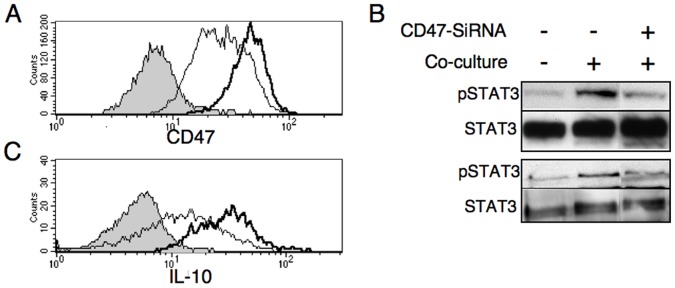
siRNA targeting CD47 attenuates STAT3 phosphorylation and IL-10 production. MCF-7 cells were transfected with either control siRNA (black line) or CD47 siRNA (grey line). (A); 24 hours later, cells were immunostained for CD47 expression. Filled grey histogram: Isotype control staining. (B); Cells transfected with either control siRNA (Middle lanes) or CD47 siRNA (right lanes) were co-cultured with either monocytes (upper panels) or DC (lower panels), for 2 h. Control cell extracts were obtained by incubating the cell and the monocytes separately and then mixing just before lysis (left lanes). Cell extracts were subjected to SDS-PAGE and immunoblotting of anti-phosphorylated STAT3 (upper panels). Anti-STAT3 immunoblotting reveals relative amounts of protein in each lane (lower panels). One representative experiment of three independent experiments is shown. (C); Monocytes were cultured alone or co-cultured with either control siRNA- or CD47 siRNA-treated MCF-7. After 24 hours, cells were harvested and IL-10 production was detected by intracellular immunostaining and flow cytometric analysis. IL-10 expression by monocytes that were gated based on their forward- and side-scatter characteristics, is shown. Filled grey histogram: monocytes cultured alone; Thick black line: monocytes co-cultured with MCF-7 transfected with control siRNA; Grey line: monocytes co-cultured with MCF-7 transfected with CD47 siRNA. Comparable results were obtained in three independent experiments.

To explore a possible role for CD47 in STAT3 activation, we used specific siRNA against CD47 for blocking its expression in MCF-7 cells. We used a combination of two different sequences of siRNAs for CD47. The effectiveness of siRNAs was shown by the remarkable decrease in surface protein levels observed by immunostaining and flow cytometric analysis (Fig. 2A). The extent of STAT3 phosphorylation was significantly lower when siRNA-treated MCF-7 cells were incubated with either monocytes or DC as compared to mock-treated MCF-7 cells co-cultured with these cells (Fig. 2B).

We have previously demonstrated that upon contact with hMSC or tumor cells APCs acquired a distinctive regulatory profile and promoted T cell unresponsiveness. This effect was accompanied by increased secretion of IL-10 but not TGF β [7] or IL-27, an IL-12-related cytokine with paradoxical pro- and anti-inflammatory effects [25]–[28] (data not shown). This induction of IL-10 expression was monocyte dependent and required direct cell:cell contact [8]. STAT3, has been previously implicated as an important regulator of the IL-10 gene by specifically binding to a single motif in the IL-10 promoter [29]. Moreover, blockade of IL-10 receptor by specific antibody partially attenuated the immunoregulatory effects on T cell activation. Notably, blockade of STAT3 signaling, specifically in APCs using two inhibitors (JSI-124, a small natural molecule that inhibits Jak2/STAT3 signaling, and the highly specific phosphorylated STAT3 peptide inhibitor that competitively inhibits STAT3 dimerization), prevented the induction of IL-10 secretion suggesting that STAT3 activation within the APC precedes IL-10 secretion [8]. Therefore, we tested the link between CD47 expression on tumor cell surface and IL-10 induction. To this end, monocytes were co-cultured with MCF-7 cells that were either control siRNA- or CD47-specific siRNA-treated, and IL-10 was visualized via intracellular staining of monocytes that were gated based on their forward- and side-scatter characteristics. As shown in figure 2C, up-regulated expression of IL-10 by monocytes upon interaction with MCF-7 requires CD47 expression on the latter, as it was significantly attenuated in monocytes interacting with anti-CD47-siRNA treated MCF-7. These results were corroborated by evaluating secreted IL-10 in the conditioned media using ELISA (data not shown). Other CD47 specific siRNAs and control siRNAs were ineffective in decreasing CD47 expression levels and had no significant effect on both STAT3 phosphorylation and IL-10 production.

**Figure 3:**
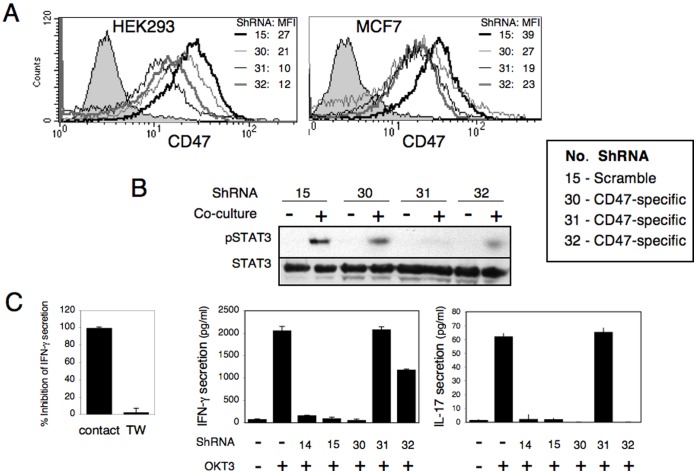
Targeting CD47 expression by shRNA abrogates STAT3 activation and restores T cell responses. HEK293 and MCF-7 cells were transfected with HuSH shRNA tRFP Cloning Vectors: clone 14: control plasmid; clone 15 plasmid containing scrambled shRNA sequence; clones 30–32: CD47 specific shRNAs. (A) Stable transfected cells were immunostained for surface CD47 expression. Numbers indicate the median fluorescent intensity (MFI) of CD47 expression in each clone. (B) The various MCF-7 transfectants were co-cultured with monocytes for 2 h (+). Control cell extracts were obtained by mixing cells and monocytes without co-culturing them together (-). Cell extracts were subjected to SDS-PAGE and immunoblotting of anti-phosphorylated STAT3 (upper panels). Anti-STAT3 immunoblotting reveals relative amounts of protein in each lane (lower panels). Comparable results were obtained in two separate experiments and similar results were obtained using HEK293 clones. (C) PBMC were incubated with or without the various HEK293 transfectants and were activated using anti-CD3 Ab (OKT3; 10 ng/ml). After 72 h, conditioned media were collected and analyzed for IFN-γ and IL-17 secretion. Left panel: to demonstrate the contact dependent inhibition, PBMC were co-cultured together or on opposite sides of a transwell and the ability of HEK293 cells to inhibit IFN-γ secretion was measured and is presented as percentage of inhibition. Right panels: The level of IFN-γ and IL-17 secreted by activated PBMC cultured in the absence or presence of the various 293 transfectants is presented. The data represent average of triplicate wells. Comparable results were obtained in two independent experiments.

To further confirm these findings, HuSH shRNA tRFP Cloning Vectors (pRFP-V-RS) expressing three different shRNA for CD47 down regulation, as well as an empty vector and vector containing a scrambled shRNA cassette used as negative controls, were transfected into HEK293 and MFC7 cells and stable lines were generated via puromycin selection. Flow cytometric analysis of cell surface CD47 expression demonstrated that two of the specific shRNA-CD47 (i.e. #31 and 32) significantly reduced CD47 levels compared with the empty vector cells (#14) or the vector containing the scrambled shRNA (#15) in both HEK293 and MCF-7 cell lines. Additional shRNA (i.e. # 30) only slightly reduced the levels of CD47 expression (Fig. 3A). To analyze the effects of CD47 knockdown on STAT3 phosphorylation, we co-incubated these various clones with monocytes for 2 h and the level of phosphorylated STAT3 was assayed as above. The extent of STAT3 phosphorylation was significantly lower when monocytes were co-incubated with HEK293 or MCF-7 clones expressing CD47 shRNA as compared to clones transfected with the control plasmids, and this correlating with the extent of decrease in CD47 expression (Fig. 3B). This effect correlated with the extent of CD47 silencing by the specific shRNA and was pronounced in clone #31. From this and the data in figure 1 it appears that when the level of CD47 decrease below 50 percent of STAT3 is not phosphorylated. These findings corroborate the link between CD47 and the contact-dependent induction of STAT3.

We have previously demonstrated that within the PMBC population, monocytes play a pivotal role in mediating hMSC and tumor cell inhibitory activity. This was exemplified in experiments where PBMC were replaced by purified CD4+ T cells and monocytes. Furthermore, inhibition of CD4+ T cell activation was observed only upon addition of either monocytes or DC to the cultures and only when the APC and hMSC were in direct contact [7], [9]. Hence, we suggested that the cell-contact mechanism conferred immunoregulatory properties for the APC population to actively suppress T cell activation, as opposed to simply being activation incompetent. In addition, blocking STAT3 signaling in the APC resulted in reversal of T cell inhibition as measured by pro-inflammatory cytokines secretion [8], [9], demonstrating that STAT3 activity in APC is linked to the induction of T cell tolerance. In order to confirm that the cell-contact mechanism that converts the APC into what is effectively an “inhibitory APC” with active immunoregulatory properties is dependent on CD47 expression, we applied T cell activation experiments. In these experiments T cells were activated with anti-CD3 in the presence or absence of HEK293 clones transfected with CD47 shRNA vectors or control vectors as described above, and IFN-γ secretion was used as a readout for T cell activation. HEK293 cells were used as opposed to cancer cells in-order to determine the exclusive contribution of the contact- (and hence CD47-) depended effect and to avoid the additional effect of soluble factor secreted by cancer cells over the 72 h period [9]. The cell-to-cell contact dependence of HEK293-mediated inhibition is based on the absence of inhibition when PBMCs and HEK293 were on opposite sides of a transwell membrane (Fig. 3C, left panel).

CD47 shRNA transfectants showed greatly reduced inhibitory activity of IFN-*γ* secretion as compared to cells transfected with the control plasmids and the extent of inhibition was inversely correlated with the level of CD47 expression on the HEK293 cells (Fig. 3C, right panels). Similar results were obtained when IL-17 secretion was tested in the conditioned media, except that IL-17 levels were recovered only after treatment of HEK293 cells with the most efficient vector, namely vector #31 (Fig. 3C, right panels). Hence, the inhibitory activity of HEK293 cells is dependent on CD47 expression, and correlated with their respective ability to induce STAT3 phosphorylation.

**Figure 4:**
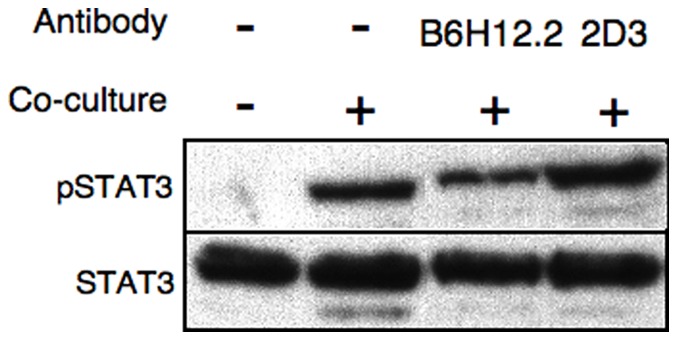
Anti CD47 antibodies targeting CD47:SIRPα interaction slightly attenuates contact-dependent activation of STAT3. MCF-7 cells were treated with either anti-CD47 blocking mAb B6H12.2 or the anti-CD47 mAb 2D3, used as control. Monocytes were co-cultured with untreated or antibody-treated MCF-7. After 2 hours, cell extracts were subjected to SDS-PAGE and immunoblotting. Anti-phosphorylated STAT3 demonstrate induction of STAT3 (upper panels). Anti-STAT3 immunoblotting reveals relative amounts of protein in each lane (lower panels). Comparable results were obtained in three separate experiments.

Next we used two monoclonal antibodies directed against CD47 [30]. The B6H12.2 mAb is capable of blocking CD47-SIRPα interaction, and the ability of this antibody to enable phagocytosis has been previously demonstrated [12]. The other isotype matched mAb (2D3) is unable to block CD47-SIRPα interaction and is used as control. MCF-7 cells were treated with these anti-CD47 mAbs and their ability to induce STAT3 phosphorylation upon interaction with monocytes was tested, as above. Surprisingly, there was relatively minor although significant decrease in STAT3 phosphorylation following B6H12.2 but not 2D3 mAbs treatment (approximately 25–30 percent reduction in band intensity; Fig. 4).

**Figure 5:**
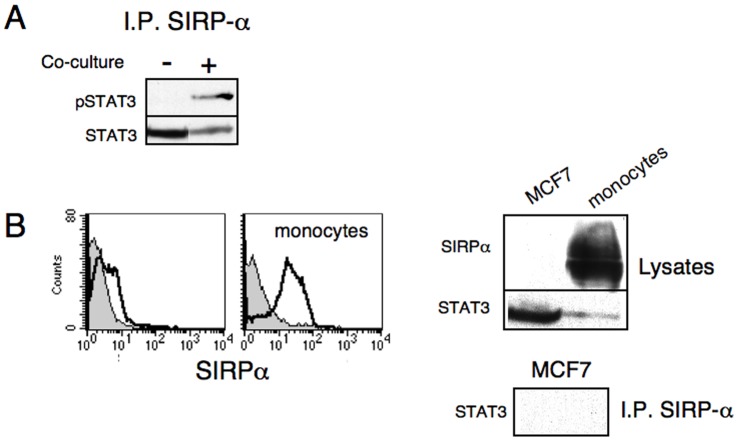
Physical association between SIRPα STAT3 and phosphorylated-STAT3. (A) Monocytes were either co-cultured with MCF-7 (+), for 2 hours or mixed with MCF-7 without incubation (-) as a control and then lysed. Cell extracts were subjected to immunoprecipitation with anti- SIRPα antibodies and the precipitates were separated on SDS-PAGE. Anti-STAT3 immunoblotting demonstrates that STAT3 associates with SIRPα (lower panel) and anti-phosphorylated STAT3 immunoblotting demonstrates association of the activated form of STAT3 upon contact with MCF-7 (upper panel). (B) monocytes and MCF-7 cells were immunostained with anti- SIRPα antibodies and then analyzed by flow cytometry (left panel). The level of SIRPα and STAT3 expression in lysates prepared from monocytes and MCF7 (right upper panels); No STAT3 was detected in anti-SIRPα precipitates from the MCF7 lysates (right lower panel). Similar results were obtained in four separate experiments.

In order to establish a link between SIRPα and STAT3, SIRPα pull-down experiments were conducted. We immunoprecipitated SIRPα from cell lysates of monocytes mixed with MCF-7 without or with 2 hours of co-culturing. Proteins in precipitates were subjected to SDS-PAGE and immunoblotting with anti-STAT3 and anti-phospho-STAT3 mAbs. Significantly, phosphorylated STAT3 was detected in anti-SIRPα precipitates upon contact-dependent activation. Furthermore, probing with anti-STAT3 revealed that STAT3 constitutively associated with SIRPα, even in the absence of cell:cell interaction and receptor triggering (Fig. 5A). To confirm that the precipitated STAT3 and phosphorylated-STAT3 originate in the monocytes and not the MSCF7 and several controls were performed. First, STAT3 was also detected in anti-SIRPα precipitates from isolated monocytes (data not shown). JSI-124 treatment (Fig. 1D) confirmed the contact-dependency of STAT3 activation, as we have previously demonstrated that this JAK2 inhibitor selectively blocks contact-dependent STAT3 activation but not that induced by soluble factors, such as IL-10 or tumor cells conditioned media [9]. Furthermore, since only monocytes were pre-treated with the inhibitor JSI-124 prior to adding them to the MCF7 cultures, the reduced level of phosphorylation of STAT3 in the co-cultures strongly suggests that this event originated in the monocytes (Fig. 1D, and [8], [9]). We also confirmed that monocytes, but not MCF-7, exclusively express SIRPα (Fig. 5B). However, while MCF7 express high levels of STAT3, no STAT3 (or pSTAT3) was detected in anti-SIRPα precipitates from MCF7 cells, used as control, corresponding to the lack of SIRPα expression in these cells Fig. 5B). In agreement with previous study that demonstrated an association between SIRPα and JAK/STAT pathway in macrophages [23], this data establishes a physical link between SIRPα and STAT3.

Taken together, these data support the notion that the CD47:SIRPα receptor:ligand pair are involved in the contact-dependent induction of regulatory APC via STAT3 activation and IL-10 production.

A key physiological circumstance that requires cell:cell contact between APC and target cell takes place during clearance of necrotic and apoptotic cells by immature DC and macrophages. Cell death by necrosis is typically associated with inflammation, in contrast to apoptosis. At the level of antigen presenting cells, it has been shown that while DC efficiently phagocytose apoptotic and necrotic cells, only the latter induce DC maturation [31], [32], phagocytosis of apoptotic cells generally suppresses maturation of professional phagocytes, and it is associated with IL-10 secretion (reviewed in [33]). On the other hand, it was suggested that on apoptotic cells, CD47 was altered and/or lost, resulting in no longer activated SIRPα, enabling apoptotic cell clearance [34].

**Figure 6:**
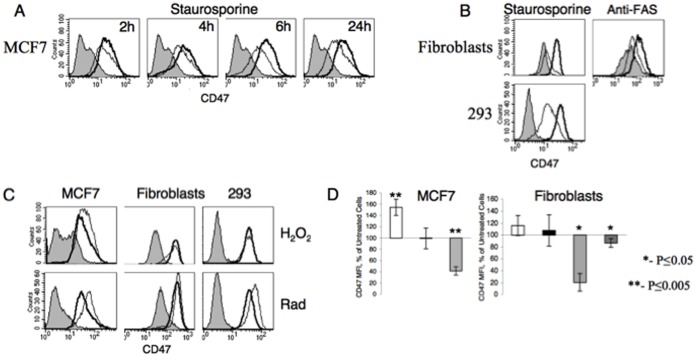
The level of CD47 expression by apoptotic cells is dependent on the mode of apoptosis induction. Apoptosis was induced by various methods as described in Materials and Methods and CD47 surface expression was measured by flow cytometry. (A) MCF-7 cells were treated with staurosporine for 1.5 h and then washed and incubated for the indicated times. Time-dependent reduction in CD47 expression is shown. (B) CD47 expression in fibroblasts (upper panels) and HEK293 cells (lower panel) 24 h after induction of apoptosis with either staurosporine (left panels) or anti-FAS (right panel). (C) CD47 surface expression on MCF-7 cells (left panel), fibroblasts (middle panel) or HEK293 cells (right panel) 24 h after apoptosis induction with H_2_O_2_ (upper panels) or γ-irradiation (lower panels). Filled grey area: isotype control; Thick black line: untreated cells; Grey line: treated cells. Similar results were obtained in at least three experiments. (D) MFI results of three separate experiments as in A-C were normalized and are presented as relative to the level of CD47 expression by live untreated cells. White bar: γ-irradiation-treated cells; Black bar: H_2_O_2_-treated cells; Light grey bar: staurosporine-treated cells; Dark grey bar: anti-FAS-treated cells. Left panel: MCF-7 cells; Right panel: fibroblasts.

Therefore, we asked whether CD47:STAT3 pathway also plays a role in the physiological circumstance of APC toleraization by apoptotic cells. To that end, we tested whether there is an apoptotic cell:APC contact-dependent mechanism, in regard to reliance on CD47 for STAT3 activation, by recapitulating with apoptotic cells what we had already performed with healthy cells. For apoptosis induction, cells were treated either with staurosporine, anti-FAS, γ-irradiation or H_2_O_2_. Apoptosis induction was verified by staining cells at various time points following treatment with annexin-V and propidium iodide exclusion followed by flow cytometric analysis. In parallel, the level of CD47 expression was tested as well (Fig. 6 A–D).

Significant decrease in CD47 surface expression in all three-cell types was observed following staurosporin treatment. This downregulation of CD47 is apparent at early stages of apoptosis even before Annexin-V is detected. Similar downregulation of CD47 was also observed in fibroblasts (the only cell type sensitive to anti-Fas) treated with anti-Fas mAb (Fig. 6 A-B and D). In contrast, CD47 expression was not reduced in cells treated with either H_2_O_2_ or γ-irradiation, and there was even a slight, but significant, increase in CD47 expression (Fig. 6 C and D).

**Figure 7:**
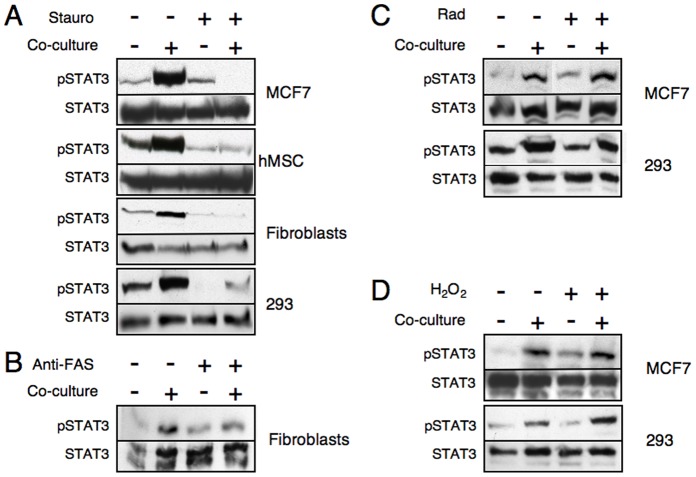
STAT3 phosphorylation in APC: apoptotic cell co-cultures correlates with CD47 expression. The various cell types were treated as depicted in figure 6. Monocytes were then co-cultured with either live or apoptotic cells for 2 hours or mixed without co-incubation as a control and then lysed. Cell extracts were separated on SDS-PAGE and anti-phosphorylated STAT3 immunoblotting (upper panels). Immunoblotting of STAT3 reveals relative amounts of protein in each lane (lower panels). (A) Staurosporine treated cells. (B) Anti-Fas treated fibroblasts. (C) γ-irradiation induced apoptotic cells. (D) H_2_O_2_-treated cells. Comparable results were obtained in four independent experiments.

The ability of apoptotic cells to activate STAT3 was directly assessed by Western Blotting using anti-phosphorylated STAT3, as described above. As seen in figure 7, apoptotic cells induced by staurosporine (Fig. 7A) or anti-Fas (Fig. 7B) do not induce STAT3 activation upon contact with monocytes, whereas, cells treated with either γ-irradiation (Fig. 7C) or H_2_O_2_ (Fig. 7D) did induce STAT3 phosphorylation correlating with their relative CD47 expression levels.

**Figure 8:**
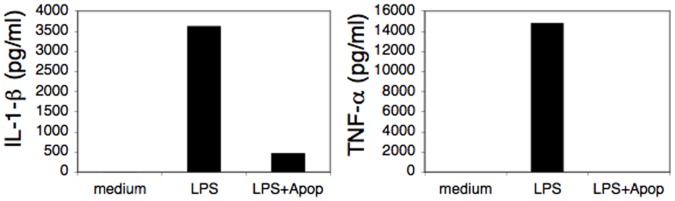
Staurosporin-induced apoptotic cells inhibit cytokine secretion by LPS-stimulated monocytes. To test whether apoptotic cells inhibit monocyte response to stimuli, monocyes were either left untreated or were stimulated with LPS in the absence or presence of staurosporin-induced apoptotic fibroblast. After 24 hours, conditioned media were collected and the levels of TNF-α and IL-1β secreted into the media were determined by ELISA. The data represent the mean values of triplicate samples and standard deviations. Data represents one of three independent experiments.

To verify that staurosporine-induced apoptotic cells regulate APC function despite their reduced CD47 expression and inability to activate STAT3, we tested their impact on APC function. As seen in figure 8, despite the decreased levels of surface CD47 in staurospoin-induced apoptotic cells and the failure to activate STAT3 in neighboring APCs, apoptotic cells significantly inhibit monocyte response to LPS. Hence, these results demonstrate that although apoptotic cells induce tolerogenic APCs in a contact dependent manner, they do not do so through the activation of STAT3 within the APC, and probably act via different mechanism.

These results demonstrate that CD47 downregulation in apoptotic cells does not take place in all modes of apoptosis and that the observed loss of STAT3 activation correlates with reduced CD47 expression in staurosporine- or anti-FAS-induced apoptotic cells. Moreover, this data suggest that in contrast to live cells, apoptotic cells (at least in certain cases) most likely use other mechanisms for toleraizing APCs.

## Discussion

Interaction of SIRPα with its ligand, CD47, is believed to signal “self” and to prevent phagocytosis of normal, healthy cells. The ligation of SIRPα by CD47 initiates inhibitory signals to phagocytes that are mediated by phosphorylation events at SIRPα’s cytoplasmic tail that contain ITIMs. These motifs recruit tyrosine phosphatases, SHP-1 and SHP-2, and initiate a signaling pathway that negatively regulates phagocytosis and macrophage activation [20], [21]. In the present study we provide evidence for a novel pathway by which CD47:SIRPα axis regulate APCs activity. In addition to the well studied ‘don’t eat-me’ signal expressed by cells via cell surface CD47 to avoid clearance by phagocytes, ligation of SIRPα on APC with CD47 expressed by neighboring cells seems also to induce STAT3 phosphorylation that converts the APCs into IL-10 secreting regulatory cells that, in turn, inhibit T cell activation.

Several lines of evidence establish the contribution of CD47 in STAT3 pathway and the conversion of APCs into regulatory cells. First, transfection of MCF-7 and 293 cells with either siRNA or shRNA against the CD47 transcript annulled the ability of these cells to induce STAT3 phosphorylation and elevated IL-10 expression by the APC. Significantly, disruption of CD47 expression resulted in a reversal of the inhibition of T cell activation. A complementary experiment demonstrated a direct physical association between SIRPα and both STAT3 and its phosphorylated form, suggesting that in addition to SHP1 and SHP2, SIRPα can also recruit STAT3 which upon cell:APC contact is phosphorylated.

Surprisingly, however, targeting cells with anti-CD47 Ab that previously has been shown to efficiently disrupt CD47:SIRPα interaction and hence enabled phagocytosis of target cells [12], had only minor effect in our assays. The fact that this Ab completely competed with the binding of a FITC-conjugated anti-CD47 antibody (data not shown), rule out the possibility of only partial blocking of available binding sites. A previous report investigated the nature of the ligand binding requirements of SIRPα and revealed two distinct but adjacent regions on SIRPα that mediate the binding to CD47 [35]. This report raises the alternative possibility (yet to be tested) that these two binding sites differ in their downstream activity. Binding of CD47 with one of the binding sites on SIRPα may lead to the recruitment of phosphatases and negative regulation of phagocytosis while interaction with the second binding region may induce STAT3 phosphorylation and regulation of APC maturation. The latter CD47 region is probably not the target of the neutralizing anti-CD47 Ab B6H12.2.

Previously, we have demonstrated that blocking STAT3 signaling in the APCs by specific inhibitors resulted in reduced IL-10 expression and reversal of hMSC and tumor cell-mediated inhibition of proinflammatory cytokines. Thus, indicating that APC’s STAT3 preceded IL-10 secretion and linking APCs’ STAT3 to the suppression of T-cell activation [8]. We further demonstrated that STAT3 activation through cell:cell contact, but not soluble factors, results in a unique APC maturation phenotype [9]. Significantly, these altered mature APC actively inhibit T cells as opposed to simply being activation incompetent [7], [9]. Taken together, we suggest an immunoregulatory circuit initiated by CD47 expressed on various somatic cells and SIRPα on DCs and monocytes that drives STAT3-dependent APC maturation arrest, IL-10 secretion, and, ultimately, T cell unresponsiveness [7]–[9].

We have initiated these studies for the purpose of studying the immunoregulatory activities of MSC and tumor cells, and demonstrated a contact-dependent mechanism for STAT3 activation that induces regulatory APC. Interestingly, here we demonstrate that this phenomenon is not limited to MSC or tumor cells, as also normal fibroblasts can similarly induce STAT3 phosphorylation upon interaction with APCs. In this regard, it is important to note that recent studies demonstrated that the ability to inhibit T cell proliferation is not restricted to MSCs, but is fundamental property of many mature, terminally differentiated mesenchymal stromal cells, such as synovial fibroblasts, dermal fibroblasts and lung-derived fibroblasts [36]–[38].

Given that CD47 is ubiquitously expressed on many cell types, the proposed mode of tolerance induction by neighboring cells through cell:APC contact may represent a more general phenomenon that is involved in peripheral tolerance in normal healthy tissues. Interestingly, CD47^−/−^ mice are viable, have only a mild phenotype, and do not develop autophagocytosis syndrome [39]. Moreover, WT macrophages that were reconstituting CD47^−/−^ mice did not respond to host cells lacking CD47. This suggests that, at least in steady state, the cellular microenvironment “educate” macrophages via the CD47-SIRPα axis [40]. Under normal physiological state, the CD47-SIRPα- dependent STAT3 activation presented in this study may provide a molecular mechanism for “educating signals” transmitted by the tissue and maintaining tolerance. The extent of STAT3 activation in local APC and hence their maturation status is determined by the level of CD47 expression in surrounding cells that, in turn, may dictate the nature of T cell response, as suggested by Matzinger and Kamala [41].

In contrast to cell death resulting from trauma that often results in inflammation and adaptive immunity, the turnover of cells in tissues during normal homeostasis seems to play a fundamental role in immune tolerance. As the cells die by apoptosis, they are rapidly engulfed by phagocytes without triggering an immune response, even tend to be anti-inflammatory, and promote tolerance [42]. While this process has been established in many studies, little is known about how precisely the apoptotic cells affect the APC that engulfs the dying cell and promote tolerance. Since the process of clearance of apoptotic cells by phagocytes requires cell:cell contact, we tested whether a similar contact-dependent mechanism for STAT3 activation also takes place in phagocyte interactions with apoptotic cells.

In the context of the ability of macrophages to discriminate between viable and apoptotic cells, it is thought that on apoptotic cells CD47 is lost, altered, or clustered into patches and can no longer activate SIRPα to generate a “don’t eat me” signal [34], [43]. As expected from this model, we found that soon after induction of apoptosis by staurosporine or anti-Fas, surface CD47 immunostaining was reduced and this correlated with their inability to induce STAT3 phosphorylation in the monocytes interacting with them. Hence, these results show that although apoptotic cells induce tolerogenic APCs in a contact dependent manner, they do not do so through the activation of STAT3 within the APC, and they probably act via a different mechanism. Surprisingly, however, cells that initiate intracellular apoptotic signaling in response to stress do not seem to downregulate CD47 surface expression, at least not immediately, and these apoptotic cells maintain their ability to induce STAT3 phosphorylation in monocytes. Therefore, we suggest that the mode by which apoptosis is induced, i.e. by extrinsic or intrinsic inducers, greatly affects the outcome of the response in regard to CD47 expression and the resulting induction of STAT3 activity in interacting phagocytes.

In this study, we have clearly demonstrated in addition to inducing a “don’t eat me signal”, CD47:SIRPα axis also induces STAT3 phosphorylation that regulates APC maturation, and, in turn, leads to T cell unresponsiveness. Hence, we suggest that CD47:SIRPα pathway can induce tolerance in more than one way. These advances in the functional analyses of the CD47-SIRPα signaling pathway and their novel role in STAT3 activation and tolerance induction in various normal and pathological cellular microenvironments may now provide exciting hints for new therapeutic targets based on manipulating this signaling pathway in autoimmune diseases.
